# Cytoprotective and Genoprotective Effects of Gelatin-Encapsulated Quercetin Against Oxidative Cell Damage

**DOI:** 10.3390/molecules31091472

**Published:** 2026-04-29

**Authors:** Alla Potapovich, Tatyana Kostyuk, Tatsiana Shutava, Vladimir Kostyuk

**Affiliations:** 1 Faculty of Biology, Department of Physiology, Belarusian State University, Nezavisimosti Ave., 4, 220030 Minsk, Belarus; pot-alla@rambler.ru (A.P.); kostyuktv@bsu.by (T.K.); 2 Institute of Chemistry of New Materials, National Academy of Sciences of Belarus, F. Skoriny St., 36, 220141 Minsk, Belarus; shutova@ichnm.by

**Keywords:** apoptosis, keratinocytes, quercetin, gelatin nanoparticles, oxidative stress, comet assay

## Abstract

The objective of this study was to compare the protective effects of native and nanostructured quercetin on the initiation of oxidative stress in human keratinocytes exposed to tert-butyl hydroperoxide (tBHP). Quercetin was encapsulated within gelatin-based nanocontainers, forming nanoparticles with diameters ranging from 140 to 180 nm. Two formulations were prepared: uncoated gelatin nanoparticles (NP1) and gelatin nanoparticles coated with a shell composed of dextran sulfate and a chitosan–dextran copolymer (NP2). Cell viability was assessed using PrestoBlue™ reagent. Apoptotic and necrotic cell populations were identified via flow cytometry using an Annexin V-FITC/PI staining kit. DNA damage was evaluated using the comet assay. The results demonstrate that gelatin nanoparticles effectively encapsulate quercetin, and the nanostructured form enables its application in aqueous suspensions without compromising its antioxidant, gene-protective, and cytoprotective effects under conditions of cellular oxidative stress. These findings suggest that gelatin nanoparticles are suitable carriers for quercetin, owing to their high aqueous solubility, which may improve its potential for oral or topical delivery.

## 1. Introduction

Oxidative stress plays a central role in the pathogenesis of numerous diseases by causing damage to cell membranes, proteins, and DNA. It is implicated in the development of cardiovascular conditions such as atherosclerosis and hypertension, as well as in the progression of neurodegenerative disorders, including Alzheimer’s and Parkinson’s diseases. Additionally, oxidative stress amplifies inflammatory processes, thereby exacerbating chronic disease progression and hindering recovery [1]. Promising therapeutic strategies for these conditions involve the use of antioxidants [2]. Plants are a primary source of natural antioxidants due to their high content of flavonoids, other polyphenols (PP), organosulfides, and indoles [3]. Several studies have demonstrated the antioxidant and anti-inflammatory effects of green tea polyphenols [4], quercetin, and resveratrol [5,6]. However, despite their biological potential, the clinical application of polyphenols is limited primarily by their poor water solubility, which results in low bioavailability when administered orally. To overcome these limitations, polyphenolic molecules can be incorporated into delivery systems such as liposomes or polymeric nanoparticles—platforms that have shown great promise for enhancing the bioavailability and stability of bioactive compounds [7,8]. Nanotechnology is an expanding field within science and biotechnology that offers new opportunities in biomedicine and cosmetology [7,9]. Previous research has demonstrated that the bioavailability of quercetin—a common flavonoid found in many foods—can be significantly enhanced by encapsulating it within microparticles featuring a chitosan/dextran sulfate shell. Importantly, microstructured quercetin retains its antioxidant activity [10]. Among various biocompatible nanocarriers, biodegradable gelatin nanoparticles are particularly promising for enhancing the cellular uptake and stability of quercetin [11,12]. In addition to their degradability, significant advantages of gelatin nanostructures include low toxicity and straightforward, cost-effective production. However, limitations in their application include potential immunogenicity and limited stability during long-term storage. Gelatin nanoparticles appear to be a promising alternative to hydrophobic nanoparticles made from corn protein zein, which are frequently used for encapsulating quercetin in experimental studies [13]. Several studies have shown that coating gelatin or other protein-based nanoparticles with polysaccharides such as dextran sulfate or chitosan derivatives enhances their resistance to gastric conditions upon oral administration [14,15]. The primary goal of this study was to evaluate the feasibility of creating water-soluble nanostructures based on gelatin, both uncoated and coated with dextran sulfate and a chitosan–dextran copolymer, aimed at increasing the bioavailability of poorly water-soluble quercetin. Since quercetin is an effective antioxidant, we utilized a cellular oxidative stress model to assess the efficacy of both free and encapsulated quercetin. This model involved keratinocytes, major cells of the skin, which is the largest organ and one of the primary targets of radical attacks from external factors.

## 2. Results

### 2.1. Physicochemical Properties of Gelatin Nanoparticles

**Figure 1:**
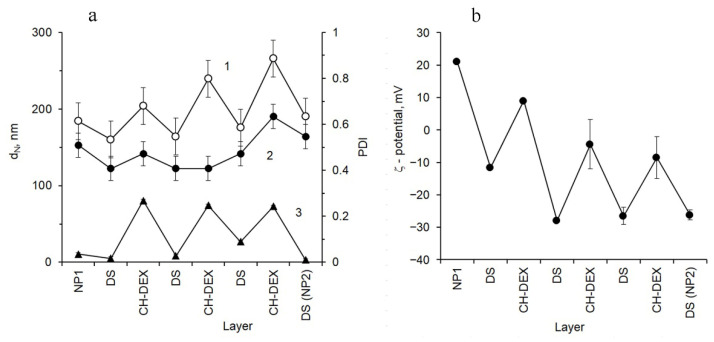
Changes in dimensional characteristics 1—Z_a_, 2—d_N_, 3—PDI (**a**) and ζ-potential (**b**) of gelatin NP 1 particles in the process of assembly of 3.5 bilayers of DS and CH-DEX on their surface to obtain LbL-coated NP2 capsules.

The average hydrodynamic diameter (Zₐ) of NP1 particles, obtained via the two-stage desolvation method and measured by dynamic light scattering (DLS), is 184 ± 20 nm (Figure 1a). The number-weighted mean diameter (d_N_) of the prepared NP1 particles is 158 ± 15 nm, which does not differ significantly from Zₐ. This consistency indicates an almost complete absence of large particles and suggests a narrow size distribution of the bare, unloaded gelatin nanoparticles. The polydispersity index (PDI) of the sample is 0.036 ± 0.006, reflecting a highly uniform particle population. NP1 are characterized by a high ζ-potential value of +21.10 ± 0.23 mV (Figure 1b). The substantial positive surface charge confers high colloidal stability to the nanoparticle suspension. During layer-by-layer (LbL) assembly with dextran sulfate (DS) and chitosan–dextran conjugate (CH-DEX), alternating changes in ζ-potential confirm the formation of a polyelectrolyte shell on the particle surface. Due to the lack of reliable visualization techniques for polyelectrolyte distribution within nanoparticles, it is presumed that upon adsorption, polyelectrolytes form layers exclusively on the particle surface. However, it cannot be ruled out that short-chain polyelectrolytes—particularly DS with a molecular weight of approximately 6 kDa—may penetrate into the soft, swollen gelatin matrix to a limited depth. This matrix consists of only about 15% gelatin by dry weight [16]. The high concentration of DS required for recharging NP1 particles supports this possibility.

An unexpected feature observed during LbL assembly is the gradual decrease in ζ-potential as additional layers of CH-DEX are deposited on NP1 particles (Figure 1b). It has been previously shown that the shell formed through LbL assembly comprises an inner layer of interpolyelectrolyte complexes and an outer hydrophilic layer composed of side chains extending outward from the surface. As more CH-DEX layers are adsorbed, this outer layer becomes denser, which can hinder further adsorption of large macromolecules due to limited space between side chains protruding perpendicularly from the surface, despite ongoing electrostatic interactions. Due to their low ζ-potential values, nanoparticles with an outer CH-DEX layer tend to partially aggregate; these particles exhibit higher Zₐ and PDI values. Subsequent adsorption of DS restores a highly negative ζ-potential, resulting in reduced hydrodynamic diameter and PDI. Consequently, gelatin nanoparticles designated as NP2 are formed with diameters only slightly larger than those of NP1.

**Figure 2:**
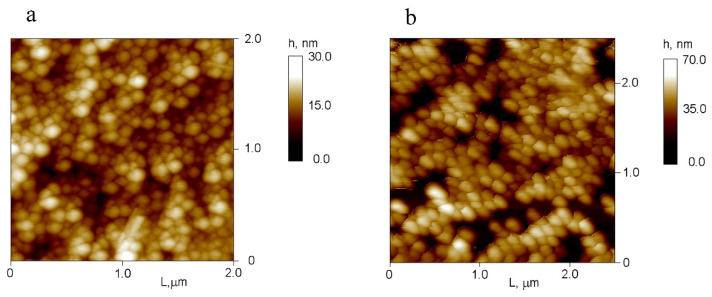
Atomic force microscopy images of Qr-NP1 on a bare silica substrate (**a**) and Qr-NP2 on a PEI/PSS/PEI-coated substrate (**b**).

Following loading with quercetin, bare gelatin particles exhibit partial aggregation, evidenced by increased Zₐ and PDI values; however, their ζ-potential remains consistently positive (Table 1). In contrast to Qr-NP1, gelatin nanoparticles coated with a (DS/CH-DEX)_3.5_ shell do not show signs of aggregation after quercetin loading. This observation aligns with previous findings demonstrating improved colloidal stability in gold and magnetite nanoparticles coated with chitosan graft copolymer shells [17]. These results confirm that adding a polyelectrolyte shell can effectively modulate nanoparticle interactions even under conditions where hydrophobic drug encapsulation tends to promote irreversible aggregation [18]. Atomic force microscopy (AFM) analysis (Figure 2) shows that the average diameters of Qr-NP1 and Qr-NP2 particles range from approximately 140 to 180 nm; their heights do not exceed 30 nm and 70 nm, respectively. Qr-NP1 particles appear more heterogeneous in size compared to Qr-NP2 in AFM images, likely due to minor losses of smaller fractions during multiple centrifugation steps involved in LbL shell formation. Additionally, differences in drying behavior between samples—with one possessing a hydrophilic shell—may contribute to this slight variation. Nonetheless, these AFM results are consistent with DLS measurements.

### 2.2. Protective Effects of Gelatin-Encapsulated Quercetin Against Oxidative Stress

**Figure 3:**
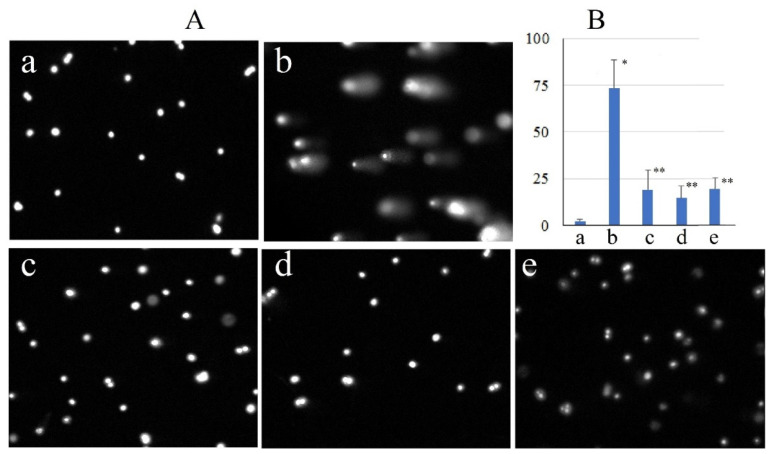
Representative fluorescence micrographs of DNA comets (**A**) and DNA content in the comet tail in percent (**B**) after 1 h of cell incubation. (**a**)—control; (**b**)—0.5 mM tBHP; (**c**)—50 μM quercetin and tBHP; (**d**)—50 μM Qr-NP1 and tBHP; (**e**)—50 μM Qr-NP2 and tBHP. *—*p* < 0.00001 vs. control: **—*p* < 0.00001 vs. tBHP.

Tert-Butyl hydroperoxide is widely used in scientific research due to its capacity to generate reactive oxygen species (ROS) within cells, thereby inducing oxidative damage to biomolecules [19]. This property enables the modeling of pathological processes characteristic of various diseases, the investigation of cellular adaptation mechanisms to oxidative stress, and the assessment of antioxidant compounds’ protective effects. In this study, exposure of human keratinocytes (HaCaT cell line) to 0.5 mM tBHP resulted in DNA damage detectable after 1 h, as confirmed by DNA comet assay. Fluorescence micrographs illustrating the electrophoretic patterns of nuclei exposed to tBHP (Figure 3A(b)) reveal typical DNA comet structures characterized by a distinct head (intact DNA) and a tail (damaged DNA). The size and fluorescence intensity of the tail varied among cells, indicating heterogeneity in DNA damage levels. After prolonged incubation to 2 h, the extent of nuclear DNA fragmentation increased, leading to the formation of atypical comets with virtually no head and a broad, diffuse tail containing over 90% of nuclear DNA. Such comet morphology is indicative of apoptosis [20]. To quantitatively evaluate the genoprotective effects of native and nanostructured quercetin, we conducted DNA comet assays on keratinocytes following 1 h of incubation with 0.5 mM tBHP in the presence or absence of 50 μM of the test compounds. The results demonstrated that both native quercetin and quercetin-loaded gelatin nanoparticles significantly altered the morphology of DNA comets in cells exposed to tBHP (Figure 3A(c–e)). Specifically, nuclei from cells treated solely with tBHP exhibited comets with prominent, diffuse tails, classified as categories “3” or “4” based on visual scoring [21]. In contrast, cells co-treated with tBHP and either native or nanostructured quercetin displayed comets with minimal or no tail formation; these were classified as categories “0” or “1.”

Quantitative analysis of fluorescence micrographs (Figure 3B) revealed that in cells exposed to 0.5 mM tBHP alone almost 75% of total nuclear DNA was localized within comet tails. Co-treatment with native or nanostructured quercetin reduced tail DNA content to less than 20%, indicating substantial protection against oxidative DNA damage.

Experimental conditions include: (I) untreated control; (II) 0.5 mM tBHP alone; (III) tBHP with 50 μM free quercetin; (IV) tBHP with 50 μM Qr-NP1; and (V) tBHP with 50 μM Qr-NP2. Quadrants in flow cytometry plots represent cell populations as percentages: lower left—live cells (Annexin V negative, PI negative); lower right—early apoptotic cells (Annexin V positive, PI negative); upper right—late apoptotic cells (Annexin V positive, PI positive); upper left—necrotic cells (Annexin V negative, PI positive). Fluorescent signals from 10,000 cells are displayed.

**Figure 4:**
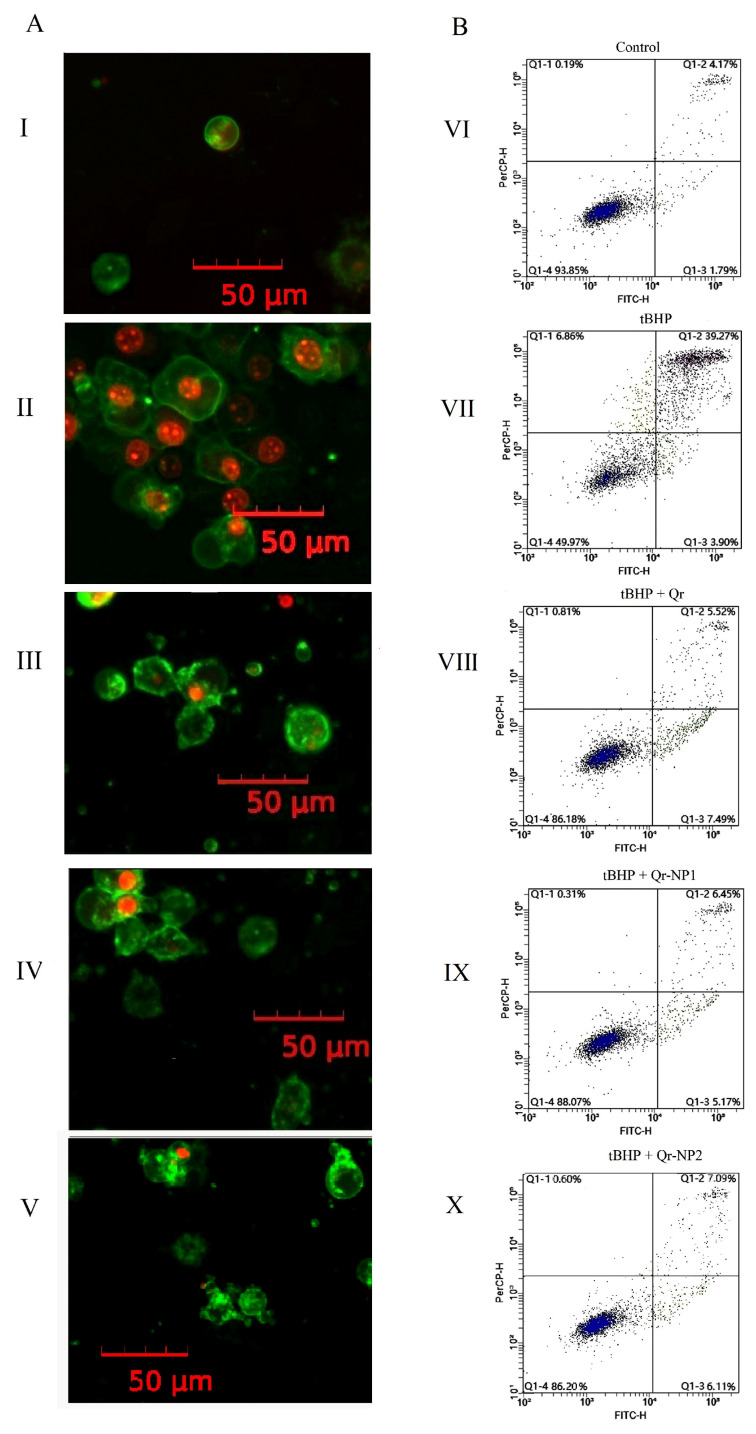
Representative fluorescence micrographs (**A**) and flow cytometry analysis (**B**) of HaCaT cells 4 h after exposure to 0.75 mM tBHP, with and without 50 μM free or nanostructured quercetin; Annexin V-FITC and PI staining. (**I**,**VI**)—control; (**II**,**VII**)—tBHP; (**III**,**VIII**)—Qr and tBHP; (**IV**,**IX**)—Qr-NP1 and tBHP; (**V**,**X**)—Qr-NP2 and tBHP; Quadrants in flow cytometry plots represent cell populations as percentages: lower left—live cells (Annexin V negative, PI negative); lower right—early apoptotic cells (Annexin V positive, PI negative); upper right—late apoptotic cells (Annexin V positive, PI positive); upper left—necrotic cells (Annexin V negative, PI positive). Fluorescent signals from 10,000 cells are displayed.

To elucidate the type of cell death induced by oxidative stress triggered by tBHP and to evaluate the protective effects of native and nanostructured quercetin, a dual fluorescence staining assay (annexin V-FITC and PI) was employed. Annexin V selectively binds to phosphatidylserine (PS), which is normally confined to the inner leaflet of the plasma membrane in viable cells. During early apoptosis, membrane asymmetry is disrupted, resulting in externalization of PS and enabling annexin V-FITC binding, which produces green fluorescence (Figure 4A(I,III–V)). In contrast, necrotic and late apoptotic cells exhibit compromised membrane integrity, allowing PI to intercalate into nuclear DNA and produce red fluorescence (Figure 4A(II)). Cell populations were analyzed by flow cytometry to assess fluorescence intensity and quantify the proportions of viable (normal), early apoptotic, necrotic, and late apoptotic cells. Following a 4-h incubation with tBHP, approximately 43% of the cell population exhibited early or late apoptosis while necrotic cells accounted for near 7% (Figure 4B(VII)). The addition of quercetin—either in its free form or as nanostructured particles, including uncoated gelatin nanoparticles or gelatin nanoparticles coated with dextran sulfate and chitosan–dextran—completely mitigated its cytotoxic effects when administered simultaneously with tBHP. Under these conditions, the distribution of cell states closely resembled that observed in control sample (Figure 4B(VI,VIII–X)).

The cytoprotective effects of both free and nanostructured quercetin against tBHP-induced oxidative stress in keratinocytes were further confirmed after 24 h of incubation over a concentration range of 12.5–50 μM (Table 2). As shown in the table, in the absence of the tested agents, the number of metabolically active cells—assessed by their ability to reduce resazurin to resorufin, a fluorescent indicator of cell viability—decreased by nearly 90%. The addition of quercetin to the culture medium, in combination with 12.5 μM tBHP, significantly increased the number of metabolically active cells. At a concentration of 25 μM, the number of viable cells after 24 h exceeded 90%. The cytoprotective activity of quercetin was fully retained when delivered via uncoated or modified gelatin nanoparticles. Multiple group comparisons revealed no significant differences between free quercetin and the nanoencapsulated forms Qr-NP1 and Qr-NP2, supporting the claim of functional equivalence between these formulations. In contrast, no protective effect was observed with gelatin nanostructures lacking quercetin.

**Figure 5:**
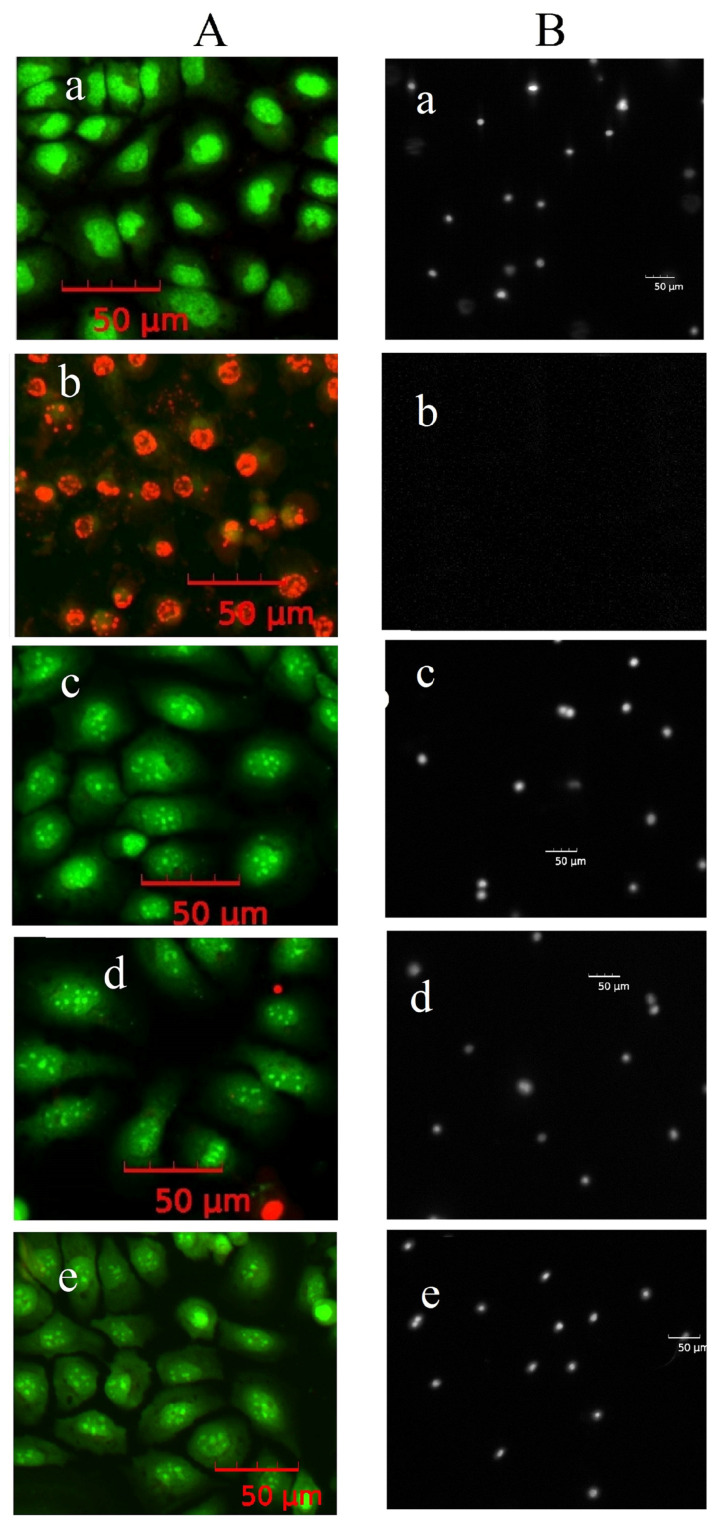
Representative fluorescent microphotographs of keratinocytes stained with the AO/EB system (**A**), and DNA comets (**B**) after 24 h of incubation with 0.5 mM tBHP. (**a**)—control; (**b**)—tBHP; (**c**)—50 μM quercetin and tBHP; (**d**)—50 μM Qr-NP1 and tBHP; (**e**)—50 μM Qr-NP2 and tBHP.

The pronounced cytoprotective effects of both free and nanostructured quercetin following 24 h of incubation with tBHP were also proved by double staining using vital fluorescent dyes—acridine orange (AO) and ethidium bromide (EB) (Figure 5A). AO stains the cytoplasm and nuclei of living cells green, whereas EB penetrates only cells with compromised membrane integrity, staining their nuclei orange or red. As shown in the images, after 24 h of incubation with tBHP, cells exhibit intense red fluorescence due to EB staining of the nuclei (Figure 5A(b)). In contrast, cells treated with free or nanostructured quercetin are stained only with AO and retain green fluorescence (Figure 5A(c–e)), indicating preserved cellular integrity. Notably, these cells display pronounced nuclear granularity. However, results from the DNA comet assay showed no evidence of DNA fragmentation capable of migrating under electrophoresis conditions to form a comet tail (Figure 5B(c–e)). The observed chromatin granularity, which reflects the degree of DNA and associated protein compaction within the cell nucleus, can be related to various cellular processes and conditions, including the cellular response to oxidative stress induced by tert-butyl hydroperoxide (tBHP) [22].

## 3. Discussion

Our data support the notion that tert-butyl hydroperoxide (tBHP) induces reactive oxygen species (ROS) formation in cells and tissues, consistent with its established pro-oxidant properties. The accumulation of ROS leads to oxidative damage of cellular components, including lipids, proteins, and nucleic acids, ultimately impairing cellular metabolism and reducing cell viability. A key mechanism underlying tBHP-induced cytotoxicity is apoptosis triggered by mitochondrial dysfunction. Specifically, elevated ROS cause mitochondrial membrane depolarization and permeabilization, leading to the release of pro-apoptotic factors such as cytochrome c into the cytosol, which then activate caspase cascades culminating in programmed cell death [23]. Without effective clearance, apoptotic cells can progress to secondary necrosis, characterized by loss of plasma membrane integrity and release of intracellular contents that may provoke inflammatory responses [24]. This pathological sequence highlights the importance of mitigating oxidative damage early in the apoptotic pathway. Quercetin, known for its potent antioxidant capabilities, appears to exert mitochondrial protection by scavenging ROS and stabilizing mitochondrial membranes, thus preventing the initiation of the intrinsic apoptosis pathway. This protection preserves mitochondrial function, DNA integrity, and overall cell viability under tBHP-induced stress [19,20,25]. Mechanistically, quercetin can modulate multiple signaling pathways related to oxidative stress, including upregulating endogenous antioxidant enzymes like superoxide dismutase (SOD) and glutathione peroxidase (GPx) [26,27] and attenuating pro-apoptotic signaling by activating the p38/Nrf2/HO-1 pathway and inhibiting the ROS/mitochondrial apoptotic pathway [27,28], which collectively preserve cellular homeostasis.

Our results demonstrate that encapsulating quercetin within uncoated gelatin nanoparticles, as well as gelatin nanoparticles coated with chitosan–dextran (CH-DEX) and dextran sulfate (DS), does not diminish its protective efficacy against tBHP-induced oxidative damage. In fact, nanoencapsulation offers potential advantages regarding bioavailability and cellular delivery. Nanoencapsulation may enhance cellular uptake via endocytosis pathways [12], protect quercetin from premature degradation or metabolism, and enable controlled intracellular release, thereby improving its therapeutic potential and cosmetic efficacy. The multilayer coating may also provide additional stability and modulate interactions with cellular membranes or the extracellular matrix, potentially improving targeting and retention in oxidative stress environments. These suggestions align with recent literature documenting improved antioxidant and cytoprotective effects of quercetin-loaded nanocarriers. For example, studies employing polymeric nanoparticles, liposomes, and nanostructured lipid carriers have reported enhanced cellular uptake, sustained release profiles, and superior protection against oxidative insults compared to free quercetin [11,12,29]. Our water-soluble gelatin-based nanosystems complement these findings by offering biocompatibility and easy functionalization, which could be further optimized to fine-tune release kinetics and target specific cell types.

## 4. Materials and Methods

### 4.1. Chemicals and Reagents

Modified Eagle’s Medium (DMEM), acridine orange (AO), ethidium bromide (EB), quercetin (Qr), tert-butyl hydroperoxide (tBHP), gelatin type A (300 Bloom) (Sigma-Aldrich, Darmstadt, Germany); isotonic phosphate buffer (IPB), pH 7.4 (Lonza, Verviers, Belgium); fetal bovine serum (FBS) (Capricorn, Świebodzice, Poland); dextran (DEX) from Leuconostoc spp., 6 kDa (Sigma-Aldrich); dextran sulfate sodium salt (DS), 6.5–10 kDa (Sigma-Aldrich); chitosan of medium molecular weight (~1250 kDa, degree of deacetylation 92%) (Glentham Life Sciences, Corsham, UK). Chitosan was depolymerized following [30] to obtain a molecular weight of approximately 20 kDa (designated as CH). Graft copolymer of chitosan and dextran (CH-DEX) with an amino group substitution degree of approximately 0.1 mol/mol was synthesized and characterized as described elsewhere [31].

### 4.2. Cell Culture

The immortalized human keratinocyte cell line HaCaT was provided by Dr. N. E. Fusenig (Heidelberg, Germany). Cells were cultured in T25 flasks (Sarstedt, Newton, NC, USA) in DMEM supplemented with 10% FBS under standard conditions at 37 °C in a humidified atmosphere containing 5% CO_2_. For experiments, cells were seeded into 96- and 24-well plates (Sarstedt, USA).

### 4.3. Preparation of Quercetin-Loaded Gelatin Nanoparticles

Gelatin nanoparticles (NP1) were prepared via a modified two-stage desolvation method without surfactants, as described by Coester et al. [11]. Compared to gelatin particles produced by other methods, the gelatin nanoparticles created via the two-step desolvation process do not contain any surfactants, exhibit low polydispersity, and are composed of approximately 85% water when suspended in an aqueous solution [15]. To produce nanoparticle capsules coated with layers of CH-DEX and DS (NP2) via layer-by-layer assembly, a thin polymeric shell was assembled on the surface of the initial gelatin nanoparticles. Specifically, DS was added in three consecutive aliquots to a ~5 mg/mL dispersion of NP1 to reach a final concentration of 1 mg/mL, inducing an alternating positive ζ-potential on the bare nano1 particles. Unadsorbed DS was removed by three centrifugation-based washes. Subsequently, layers of CH-DEX and DS (~0.5 mg/mL polyelectrolyte solutions) were deposited sequentially onto the nanoparticle surface through electrostatic interactions. The resulting nanocapsules were redispersed in water. To load quercetin into gelatin nanoparticles, dispersions of bare or coated nanoparticles were diluted to 6.5 mg/mL based on dry weight. A quercetin solution in ethanol at 1 mg/mL was added dropwise under constant ultrasound treatment to achieve a final Q concentration of approximately 45 μg/mg nanoparticle mass; this resulted in a final ethanol volume fraction of about 30%. The mixture was incubated for 30 min at room temperature. Quercetin-loaded nanoparticles (Qr-NP1 and Qr-NP2) were then separated by centrifugation and redispersed in distilled water. Quercetin loading efficiency was determined spectrophotometrically by extracting Qr with ethanol and measuring absorbance at 378 nm.

### 4.4. Characterization of Nanoparticles

Hydrodynamic diameters and polydispersity index (PDI) were measured using dynamic light scattering (DLS) with a Zetasizer Nano ZS instrument (Malvern Instruments, Malvern, UK). ζ-Potential values were obtained via electrophoretic light scattering on the same device. Morphology was examined using atomic force microscopy with a MultiMode Nanoscope III system equipped with NPS-1 silicon nitride probes; images were processed using Nanoscope v5.31 software.

### 4.5. Induction of Oxidative Stress and Cell Viability Assays

Oxidative stress was induced by adding tBHP to cell cultures. Quercetin and aqueous suspensions of nanoparticle-encapsulated quercetin were added immediately prior to tBHP treatment in serum-free DMEM medium. Stock solutions of quercetin (10 mM) were prepared in DMSO. In all experiments, the DMSO concentration in the medium was 0.5%. Cell viability was assessed after 24 h using PrestoBlue™ reagent (Introvigen, Waltham, MA, USA) according to the manufacturer’s instructions. Fluorescence intensity was measured at excitation/emission wavelengths of 560/590 nm using a Clariostar Plus spectrofluorometer (BMG Labtech, Ortenberg, Germany, Germany). Fluorescence readings from control wells were set as 100%. Data from three independent experiments were averaged for each experimental condition.

### 4.6. Assessment of Cell Integrity and Apoptosis/Necrosis

Cell membrane integrity was evaluated via live/dead staining in the wells of a 24-well plate without removing the cells. Adherent cells were first washed with PBS, after which 0.25 mL of PBS containing 2.5 μL of AO/EB dyes (AO at 3 mg/mL and EB at 3 mg/mL) was added to each well. Following a 15-min incubation in the dark, cell staining was observed using a fluorescence microscope (Celena S Digital Cell Imaging System, Logos Biosystems, Anyang, Republic of Korea). Apoptotic and necrotic cells were identified through double vital staining with annexin V-FITC and propidium iodide (PI) using an assay kit from BioLegend (San Diego, CA, USA), according to the manufacturer’s instructions. Cells stained for apoptosis/necrosis were analyzed by flow cytometry on a LongCyte^®^ CE instrument (Beijing Challen Biotechnology Co., Ltd., Beijing, China) or visualized via fluorescence microscopy coupled with the Celena S Digital Cell Imaging System.

### 4.7. DNA Damage Analysis via Comet Assay

DNA strand breaks were quantified using the alkaline comet assay as described by Tice et al. [32]. Briefly, cell suspensions were embedded in agarose-coated slides and lysed overnight at 4 °C in lysis buffer in darkness. Slides were then immersed in alkaline buffer solution (pH > 13; containing NaOH and EDTA) for DNA unwinding for 20 min at room temperature. Electrophoresis was performed at 300 mA for 20 min under alkaline conditions. After electrophoresis, slides were fixed in ethanol and stained with EB. Comet images were captured using fluorescence microscopy combined with the Celena S Digital Cell Imaging System. The percentage of DNA in the tail (% tail DNA), indicative of DNA damage, was quantified using ImageJ software (1.54g). Data from three independent experiments were averaged for each experimental condition (n ≈ 150 cells).

### 4.8. Statistical Analysis

All data are expressed as mean ± standard error (SE). Statistical comparisons between two groups were performed using a two-tailed unpaired *t*-test. Multiple group comparisons were conducted using one-way ANOVA followed by a post hoc Tukey–Kramer multiple comparisons test. A *p*-value < 0.05 was considered statistically significant. All analyses were performed using GraphPad Prism version 5 (GraphPad Software, La Jolla, CA, USA).

## 5. Conclusions

This study employed a tert-butyl hydroperoxide-induced oxidative stress model to assess the potential of both uncoated and coated gelatin nanoparticles as delivery systems for enhancing the bioavailability and efficacy of phytochemicals, particularly quercetin. Encapsulation of quercetin within these nanoparticles enabled its application in aqueous suspensions without compromising its antioxidant, genoprotective, and cytoprotective activities under oxidative stress conditions. Both free and nanoparticle-encapsulated quercetin similarly protected keratinocytes from oxidative DNA damage, necrosis, and apoptosis induced by tBHP. This effect may be attributed to the efficient release of quercetin from uncoated gelatin nanoparticles as well as those coated with layers of CH-DEX and DS. Additionally, surface modifications can improve nanoparticle resistance to gastrointestinal conditions and facilitate targeted delivery. Overall, gelatin nanoparticles represent a promising platform for enhancing the bioavailability and therapeutic potential of phytochemicals, advancing the application of nanotechnology in pharmaceutical development.

Both free quercetin and nanoparticle-encapsulated quercetin provided similar protection to keratinocytes against oxidative DNA damage, necrosis, and apoptosis induced by tBHP. This effect may be attributed to the efficient release of quercetin from uncoated gelatin nanoparticles as well as those coated with layers of CH-DEX and DS.

## Figures and Tables

**Table 1:** Characteristics of gelatin-based nanoparticles used for biological activity tests.

Nanoparticles	Z_a_, nm	PDI	d_N_, nm	ζ-Potential, mV	C_Q_, mmol/g
NP1	205	0.033	190	22.6 ± 1.6	0
Qr-NP1	308	0.430	190	19.3 ± 2.8	0.175
NP2	198	0.087	141–164	−26.2 ± 0.9	0
Qr-NP2	201	0.076	164–190	−21.0 ± 4.0	0.217

**Table 2:** Effect of free and nanostructured quercetin on the number of metabolically active cells (as a percentage of control) after 24 h of incubation with 0.5 mM tBHP.

Samples	Concentration of Qr in the Medium (μM)			
	0	12.5	25	50
tBHP and Qr	12.0 ± 7.1 ^§^	59.7 ± 12.4 ^¥^	92.2 ± 7.0 ^¥¥^	86.0 ± 6.3 ^¥¥^
tBHP and Qr-NP1	14.8 ± 10.6 ^§^	61.2 ± 15.8 ^¥^	92.6 ± 5.8 ^¥¥^	92.4 ± 10.0 ^¥¥^
tBHP and Qr-NP2	14.8 ± 10.6 ^§^	ND *	82.6 ± 6.4 ^¥¥^	91.4 ± 3.6 ^¥¥^

^§^—*p* < 0.000001 vs. control; ^¥^—*p* < 0.001; ^¥¥^—*p* < 0.0001 vs. tBHP; *—ND, not determined.

## Data Availability

Data available on request due to restrictions.
